# Proof-of-Concept of Electrical Activation of Liposome Nanocarriers: From Dry to Wet Experiments

**DOI:** 10.3389/fbioe.2020.00819

**Published:** 2020-07-23

**Authors:** Laura Caramazza, Martina Nardoni, Annalisa De Angelis, Patrizia Paolicelli, Micaela Liberti, Francesca Apollonio, Stefania Petralito

**Affiliations:** ^1^ICEmB at DIET, Sapienza University of Rome, Rome, Italy; ^2^Center for Life Nano Science@Sapienza, Istituto Italiano di Tecnologia, Rome, Italy; ^3^Department of Drug Chemistry and Technologies, Sapienza University of Rome, Rome, Italy

**Keywords:** nanosecond pulsed electric fields, liposome vesicles, controlled release, electroporation, electropermeabilization, exposure systems, multiphysics modeling

## Abstract

The increasing interest toward biocompatible nanotechnologies in medicine, combined with electric fields stimulation, is leading to the development of electro-sensitive smart systems for drug delivery applications. To this regard, recently the use of pulsed electric fields to trigger release across phospholipid membranes of liposomes has been numerically studied, for a deeper understanding of the phenomena at the molecular scale. Aim of this work is to give an experimental validation of the feasibility to control the release from liposome vesicles, using nanosecond pulsed electric fields characterized by a 10 ns duration and intensity in the order of MV/m. The results are supported by multiphysics simulations which consider the coupling of three physics (electromagnetics, thermal and pore kinetics) in order to explain the occurring physical interactions at the microscopic level and provide useful information on the characteristics of the train of pulses needed to obtain quantitative results in terms of liposome electropermeabilization. Finally, a complete characterization of the exposure system is also provided to support the reliability and validity of the study.

## Introduction

In the last few decades, there has been great interest in developing drug delivery systems involving the use of liposomal nanodevices carriers, as promising tools either for treatment of cancer diseases (Rosenblum et al., 2018; Senapati et al., 2018; Riley et al., 2019) or for non-cancer ones such as cardiovascular, neurological and autoimmune disorders, respiratory system diseases, skin illness (Bayat et al., 2020; Moncalvo et al., 2020). Liposomes are like toolboxes of lipids which can be manipulated, tuned and manufactured to demand in order to control their biochemical characteristics in terms of dimension, composition and drug release rate. Since the pioneering research of Bangham and Horne (1964), liposomes have attracted great attention in the field of drug delivery for their excellent biocompatibility (Williams, 2008), biodegradability, almost no toxicity, low immunogenicity, ability to incorporate hydrophilic and hydrophobic agents, controlled release properties, high stability, improved therapeutics efficacy, reproducible scale-up and manufacturing (Akbarzadeh et al., 2013; Allen and Cullis, 2013; Bozzuto and Molinari, 2015).

From the first generation to date, conventional liposomes were typically composed of phospholipids and cholesterol. The novel generation of lipid vesicles is based on: (i) functionalized surface to specifically reach the selected cell or tissue – ligand targeted liposomes – (Daeihamed et al., 2017), (ii) deformed and elastic structure in order to be administered via transdermal and oral routes – transfersome – (Bayat et al., 2020), and (iii) ability to be triggered by an external or internal stimulus such as pH, temperature, redox potential, enzymes, electrolyte concentration and ultimately even magnetic fields to achieve a spatiotemporal control of drug release – smart delivery system – (Murdan, 2003; Spera et al., 2014, 2015; Kim and Lee, 2017; Nardoni et al., 2018; Liu and An, 2019; Yuba, 2020). This last characteristic is particularly promising in the evolution of liposome technology, because one can think of electromagnetic fields as actuators; in practice it will allow engineers to design a rational and remote control of the release thus making liposome vesicles a reservoir of the drug or molecule to be released on site and on-demand.

Recently, an attempt to evaluate a stimulus-dependent response of giant unilamellar lipid vesicles (GUV) using a series of electric field pulses of micro- to millisecond duration, has been demonstrated to be successful in electrodeformation, electrofusion and electroporation in the membranes of these vesicles (Karal et al., 2019). Authors proved that the results of pore formation at different electrical-induced membrane tensions are in agreement with those reported for mechanical-induced ones. Similar results have been reported in Perrier et al. (2018), where it was observed an ejection of fluid-phase lipids concomitant with a GUV size decrease.

In spite of the interesting and promising results, the electric fields used in these works are those typical of irreversible electroporation which, when applied to cells or tissues, imply the creation of permanent and hence lethal nanopores in the cell membrane disrupting cellular homeostasis thus causing cell death. A more versatile and powerful approach could be to use electric field pulses not disrupting the cells to which they are applied. This is the well-known electropermeabilization process, which is based on the creation of transient pores in the phospholipid bilayer. Nowadays, this is becoming a technology platform for enhancing the transmembrane transport of drugs, genetic materials, and other molecules in the areas of medicine, food processing, and in some environmental applications (Kotnik et al., 2015; Perrier et al., 2017).

In particular nanosecond pulsed electric fields (nsPEFs) seem to be promising in this sense; the challenge in the application of nsPEFs relies in the possibility to cause both the cells and the liposomes membranes electroporation without triggering irreversible damage of cells. In such a way, liposome poration could permit the drugs release in the extracellular medium, close to the cells, and an easy uptake of the drugs by the electroporated cells could be achieved. Thanks to the similarity between cellular and liposomal membranes (Breton et al., 2012, 2015; Portet et al., 2012) and considering the second-order model of induced transmembrane voltage (Postow and Polk, 1996; Kotnik and Miklavčič, 2000; Merla et al., 2012), such pulsed electric fields with high frequency content could be used as a promising external trigger to obtain simultaneous and reversible electropermeabilization of both cell and liposome membranes, thus overcoming the limitation of the well-known transmembrane dependence on the radius of the microscopic structures for pulses with a lower frequency spectral content. Authors have already shown through a theoretical study the possibility of nsPEFs to induce a simultaneous electroporation of cell and liposomes using small unilamellar lipid vesicles of the order of hundreds of nm (Denzi et al., 2017a, b).

In this paper we experimentally prove that nsPEFs can trigger the release of a fluorescent dye used as a hydrophilic model drug molecule contained in the aqueous core of unilamellar liposomes, using a fully characterized and controllable experimental bench. The real-time monitoring of the dye fluorescence gives direct information on the concentration of the compound escaped from the vesicles and hence provides an estimate of the increased permeability of the lipid membrane induced by nsPEFs application.

The setup of the experiments was accompanied by its full characterization in order to obtain complete reliability and control of the experimental data. The measurements involved both the nsPEFs generation and delivery, and the temperature acquisition during the experiments; the same characterization has been obtained via simulations, ensuring the same operative exposure conditions for the liposome samples. The experimental results obtained on liposomes permeabilization are further investigated by multiphysics simulations which, exploring these physical interactions, represent a guide to achieve quantitative results when exposing nanosized liposomes to nsPEFs. This study is aimed at establishing a powerful tool to predict the results of advanced experiments on modulating nsPEFs parameters. Thus, starting from these combined numerical and experimental outcomes, future *in vitro* experiments could be performed first on cells and then on both liposomes and cells to experimentally investigate compounds release from liposomes and their uptake inside cells.

## Materials and Methods

### Materials

Egg phosphatidylcholine (Egg-PC) Lipoid 80 E from Lipoid GmbH (Germany) was kindly offered by AVG Srl (Italy). 4-(2-hydroxyethyl)-1-piperazine ethanesulfonic acid (HEPES), 5-(6) carboxyfluorescein [5-(6) CF], Triton X-100 (TX-100), Sephadex G-50 medium grade, hydrochloric acid (HCl), ammonium thiocyanate and sodium hydroxide (NaOH) were purchased from Sigma Aldrich (Italy). Chloroform (CHCl_3_) was obtained from Merck (Italy). Bidistilled water, sodium chloride (NaCl), ethanol, thiocyanatoiron and 1,2-dichloroethane were supplied by CARLO ERBA Reagents (Italy). Cyclopore polycarbonate membrane filters Whatman^®^ were purchased from Cyclopore Track Etched Membrane.

### Liposome Preparation and Characterization

According to the thin film hydration method (Petralito et al., 2012), Egg-PC was dissolved in a round bottom flask containing 3 mL of CHCl_3_. The organic solvent was evaporated under reduced pressure until a thin lipid film was formed on the flask bottom, using a rotavapor. Any trace of solvent was further removed keeping the flask under reduced pressure for 2 h. The dry lipid film was then hydrated with 5 mL of the 5-(6) CF dye solution, 50 mM in HEPES buffer (σ = 0.0304 ± 0.0041 S/m, 10 mM, pH = 7.4). The buffer properties have been chosen in accordance with the literature on electroporation (Silve et al., 2016; Denzi et al., 2017b). Five consecutive freezing/thawing cycles in a dry ice-ethanol bath were performed to increase the trapped volume of multilamellar preparations. The obtained vesicles were downsized by sequential extrusion to form unilamellar liposomes. This step was performed through polycarbonate membrane filters of decreasing pore size (0.8–0.4 μm) up to five times in order to obtain a narrow size distribution. To remove the unencapsulated fluorescent dye, the sample was subjected to a size exclusion chromatography (SEC) with a Sephadex G-50 column. After the preparation, a physico-chemical characterization has been carried out in terms of both sizing (hydrodynamic diameter and size distribution) and ζ-potential, using a Zetasizer Nano ZS 90 (Malvern Instruments Ltd., Malvern, United Kingdom), at the constant temperature of 25°C, in order to ensure homogeneous size distribution and shape, with good time stability.

### Evaluation of Liposome Membrane Permeabilization

The effect of nsPEFs application on the liposomal vesicles membranes was evaluated through the carboxyfluorescein release method relying on the de-quenching of the encapsulated hydrophilic marker 5-(6) CF from the inner compartment of the liposomes to the bulk of the suspension.

When a defect forms in a liposome containing internal 5-(6) CF, its release is detected quantitatively as an increase in fluorescence. The fluorescence intensity measurements were performed in time drive modality scanning the samples with a spectrofluorometer (LS 50B Perkin Elmer, United States), using the λ_ex_ and λ_em_ maxima (λ_ex_ = 492 nm and λ_em_ = 512 nm) previously determined, up to 2 h and 30 min after the exposure. At the end of the entire set of measurements, the liposomal vesicles were completely destroyed by adding a lytic concentrated solution of the non-ionic detergent TX-100 (30% w/V) in order to evaluate the total amount of 5-(6) CF entrapped. The percentage release of the hydrophilic dye was calculated with the following equation:

(1) $$5-\left(6\right)\mathrm{CF}\mathrm{released}\left(\%\right)=\frac{\text{I}\left(\text{t}\right)-\text{I}\left({\text{t}}_{\text{ref}}\right)}{\text{I}\left({\text{t}}_{\text{max}}\right)-\text{I}\left({\text{t}}_{\text{ref}}\right)}\times 100$$

where, I(*t*_ref_) is the initial fluorescence intensity of the dye in the bulk suspension, I(t) represents the fluorescence intensity recorded at each sampling time and I(*t*_max_) corresponds to the maximum intensity after the lysis of the liposomes with TX-100. All the measurements were performed in triplicate and the results are reported as mean ± standard deviation.

### nsPEFs Exposure Setup: Experimental Characterization and Numerical Modeling

**FIGURE 1:**
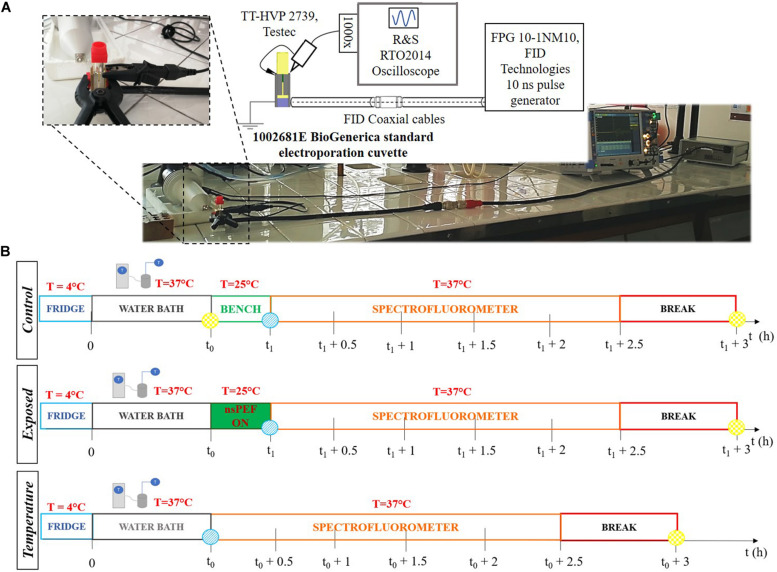
**(A)** Exposure setup configuration and picture of the laboratory equipment used to perform the experiments. The generator is connected through two coaxial cables and a transition to the cuvette. The cuvette is filled with the liposomal suspension sample. The signal at the load is monitored in time with the R&S oscilloscope and two Testec HV probes. **(B)** Experimental protocol tuned to expose liposomal vesicles to a sequence of pulses, with a nanosecond duration and intensities in the order of MV/m. The three timelines represent the procedure performed for each sample. The *t*_0_ = 0.5 h is the thermal equilibration period, while *t*_1_ = (*t*_0_ + 0.28 h) is the start of the exposure. The times t_0_ and (*t*_1_ + 3 h) are, respectively, the reference time (blue point) and the maximum fluorescence time (yellow point) used to evaluate the electrical signal effect on the liposomes. The starting time for fluorescence analysis is *t*_1_.

The system used to perform the nsPEFs exposure was composed by a standard electroporation cuvette (1002561E, BioGenerica, Italy), hosting a fixed volume of the prepared liposomal suspension, placed in the 11 mm-gap between the two brass electrodes of the cuvette holder, that are suitable connected to a HN coaxial cable, as first designed and developed in Denzi et al. (2017b). To mechanically stabilize the structure, avoiding any air gaps between the holder and the standard cuvette, they were attached with a clamp. This exposure system was then completed with a second HN coaxial cable that was connected to the FID generator (FPG 10-1 NM10, FID Technologies, Germany) (see Figure 1A). The 10 ns pulses produced by the generator were delivered to the liposomal suspension placed in the 1 mm-gap cuvette electrodes, reaching electric field amplitudes in the order of MV/m.

The electrical signal delivered by the pulse generator was recorded using an oscilloscope (RTO2014, Rohde & Schwarz, Germany) connected to the source through a chain of four attenuators. Moreover, the electrical signal reaching the cuvette was monitored in time during each experiment. In particular, the voltage established between the two electrodes of the cuvette was measured using two HV-probes (TT-HVP 2739, Testec, Germany) connecting the exposure structure to the oscilloscope. The HV probes are characterized by a band width ranging from DC to 220 MHz and a rise time of 1.6 ns. The exposure setup is shown in Figure 1A. The electric field applied to the sample is given by the ratio of the voltage at the electrodes and the 1 mm distance.

The temperature of the sample placed in the cuvette has been monitored during the exposure using an infrared (IR) thermal camera A325 (FLIR Systems, United States) which has a thermal sensitivity <0.07 at 30°C. This camera is able to measure the temperature in the range −20 to 120°C with an accuracy of ±2%. The thermal images are composed by over 76000 individual picture elements, with a frame rate up to 60 Hz. The use of this thermal camera allows the acquisition of both thermographic images and non-contact measurements of the temperature, avoiding any interactions with the analyzed sample. A FLIR Research IR software is used both to control the real-time thermal analysis and to post-elaborate the recorded data.

Numerical modeling of the exposure setup has been investigated in order to support the experimental data and to give theoretical support to the whole procedure. The electric field distribution in the cuvette containing the buffer and the temperature profile in time during the nsPEFs was solved using the software COMSOL Multiphysics v. 5.3. A 3D model of the exposure system with realistic geometrical dimension was implemented. The cuvette was filled with a solution characterized by a relative permittivity of 85 and a conductivity of 0.03 S/m, as the dielectric properties of the HEPES buffer solution. The conductivity value of the buffer solution was confirmed with measurements with a Precision LCR Meter E4980A from Agilent, as in Denzi et al. (2017a). The multiphysics problem was solved by coupling two modules, in a Time Dependent study. The quasi-static electromagnetic problem was solved using the AC/DC modules in Electric Currents mode. The square pulse excitation, with 10 ns duration and 2 ns rise/fall time, was applied to the cuvette electrode, the brass electrode and the central pin, connected together. The ground terminal was set to the external sock and to both the cuvette and the second brass electrode connected together. Temperature increase in the buffer solution due to the electromagnetic signal, has been evaluated using the electromagnetic heating module in the Joule heating mode, starting from 25°C as initial room temperature value of the suspension.

### Experimental Protocol

Liposomal samples were prepared 24 h before the experiments and they were stored overnight in a refrigerator at 4°C. As reported in Figure 1B, the experimental protocol was characterized by the following steps: (1) thermal equilibration at 37°C of the sample up to *t*_0_ = 30 min using a cryostat water bath; (2) application of the electrical signal (nsPEFs ON) placing the sample in the electroporation cuvette for a period of time of about 17 min at room temperature (ending at *t*_1_ = *t*_0_ + 17 min); (3) analysis of the fluorescent dye release: during this step the sample is placed in a quartz cuvette and accommodated in the spectrofluorometer at the controlled temperature of 37°C and the fluorescence measurements were performed monitoring in time up to 2.5 h after the exposure; (4) complete lysis of the liposomes in order to evaluate the maximum fluorescence of the dye entrapped in the sample. The exposures have been carried out using the protocol parameters reported in Table 1, thus setting the generator at 9 kV and a pulse repetition frequency (PRF) of both 2 and 4 Hz for a period of time of about 17 min, thus exposing the liposomal sample to 2000 and 4000 pulses, respectively. Exposure parameters such as number of pulses, period of exposure and PRF have been chosen considering the literature (Silve et al., 2014; Lamberti et al., 2015). For the control samples procedure in step (2) the control was placed on the bench near the exposure system, while for the temperature control the operating temperature has been set to 37°C. Eight independent exposure experiments have been performed with 2000 pulses, and four experiments with 4000 pulses, each exposed sample has been tested simultaneously with its control.

**TABLE 1:** Operative conditions of the exposures for a dose-effect electropermeabilization evaluation.

**Electrical Parameters**					
# set-up	# nsPEFs	PRF (Hz)	Voltage (kV)	FWHM (ns)	Rise/Fall time (ns)
1	2000	2	9	10	2
2	4000	4	9	10	2

### Numerical Simulation of Liposome Membranes Electropermeabilization

**FIGURE 2:**
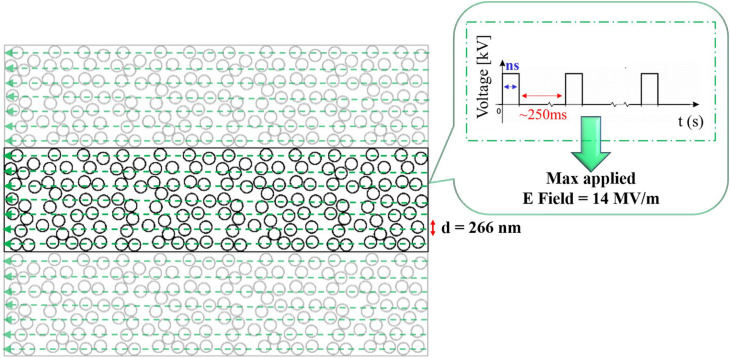
2D geometrical model of a randomly packed distribution of 156 liposomes, characterized by a diameter of 266 nm. The signal applied to the liposomes is reported in the inset and it represents the experimental train of nsPEFs delivered by the ns generator, each pulse is characterized by a 10 ns duration and 14 MV/m as maximum electric field amplitude. On the top and bottom boundaries periodic conditions were set, thus the simulation box is ideally replicated.

2D numerical simulations have been performed to model a mixture of liposomes in solution with COMSOL Multiphysics software v. 5.3. Figure 2 displays the 2D rectangular box representing the external buffer medium, 2.30 μm in width and 9.34 μm in height, where a random distribution of liposomes of 266 nm in diameter and a membrane thickness of 5 nm were placed, according to the experimental liposomes dimension. The distribution of 156 liposomes was obtained by a random distribution program built in MATLAB, avoiding the superposition of vesicles. Periodic conditions were set on the top and bottom of the box. The simulations were performed considering the left side of the box as the ground electrode and the right one excited by a train of pulses of 10 ns duration and rise/fall times of 2 ns, spaced in time of 250 ms to simulate the liposome exposure to a train of pulses delivered at 4 Hz.

The quasi-static electromagnetic problem was solved in the Electric Currents mode and coupled with two other modules. Boundary conditions were imposed on liposome membranes as contact impedance and the formation of pores on the membranes was studied solving in the Boundary ODEs and DAEs mode the asymptotic equation, firstly proposed by DeBruin and Krassowska (1998), and used in literature (DeBruin and Krassowska, 1999; Neu and Krassowska, 1999; Mercadal et al., 2016; Caramazza et al., 2019). Specifically, the pore formation across a membrane is determined by the transmembrane potential induced by the electrical stimulus. In accordance with the asymptotic model, the membrane conductivity changes are also determined by a series of parameters i.e., pore conductivity as reported in studies on synthetic membranes (He and Kyu, 2016; Liu et al., 2018; Zhang et al., 2019), transmembrane potential (TMP) and temperature, as reported in DeBruin and Krassowska (1999), Retelj et al. (2013). To this regard, the temperature distribution in the simulation box was evaluated in time by coupling the Heat Transfer in fluid module with the AC/DC module. A thin layer condition was set on liposome membranes to take into account their thermal properties and behavior. In Table 2 the material properties that were set in the simulations are reported according with the experimental data and literature (Merla et al., 2012; Caramazza et al., 2019). All the parameters used for the pore kinetics model are reported in Mercadal et al. (2016) and Retelj et al. (2013).

**TABLE 2:** Dielectric and thermal properties used to represent each material compartment in the microdosimetric simulations.

Material properties	External medium	Membrane	Internal medium
**σ [S/m]**	0.03	1.1 × 10^–7^	0.35
**ε_r_**	85	11.7	85
**Cp [J/(kg K)]**	4185.5	2000	4185.5
**ρ [kg/m^3^]**	993.2	951.1	993.2
**k [W/(m K)]**	0.62	0.2	0.62

### Statistical Analysis

All the reported data came from measurements done at least in triplicate for each sample of a single experiment. All statistical analyses were performed with one-way ANOVA to determine significant differences in the experimental data. The maximum *p*-value that was considered statistically significant was 0.05. As regards the outcomes of the signal characterization with HV probes, a program has been built in MATLAB software to analyze the sequence of pulses in terms of rise and fall times, duration and amplitude of each pulse of the delivered sequence, determining the mean values and the standard deviations.

## Results

Physicochemical features of the obtained liposomes with a diameter of 267.91 ± 1.71 nm are reported in Supplementary Table S1, showing hydrodynamic diameter, size distribution, ζ-potential, entrapment efficiency, structured phospholipid in vesicles. All measurements were performed at a scattering angle of 90° and were thermostatically controlled at 25°C. The samples were opportunely diluted with 10 mM HEPES (pH = 7.4) as in Petralito et al. (2012).

### Electrical Activation of the Release by nsPEFs

**FIGURE 3:**
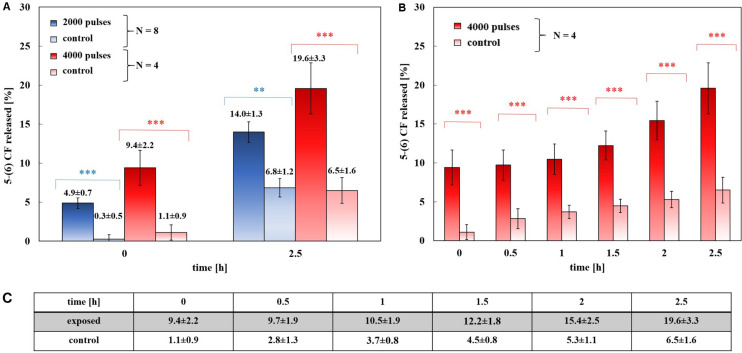
Experimental release of the 5-(6) CF in percentage. **(A)** Dose-effect evaluation: 8 trails of exposed samples at 2000 pulses at 2 Hz train (blue) and 4 trails of exposed samples at 4000 pulses at 4 Hz train (red), with their controls (light blue and light red, respectively), at 0 and 2.5 h after the exposure. The differences between the exposed samples and their control are always statistically significant with a *p* < 0.01 or *p* < 0.001. **(B)** The results of a set of four exposures using 4000 pulses and a PRF of 4 Hz and their controls, (8 samples). The release of the 5-(6) CF is shown at 0, 0.5, 1, 1.5, 2, and 2.5 h after the exposure, values reported in panel **(C)**. At each time the difference between the exposed and the control values is significant with a *p* < 0.001. **(C)** Values of 5-(6) CF experimental release at the selected times for both 4000 pulses exposed samples and the controls.

Figure 3A reports the major result of this study showing a significant increase in both exposure conditions, just afterward exposure and, then, after 2.5 h. According to the established experimental protocol, the release profile of the 5-(6) CF dye was studied after the nsPEFs stimulation of the sample and then the results were compared with the related controls, with no electrical signal application.

**FIGURE 4:**
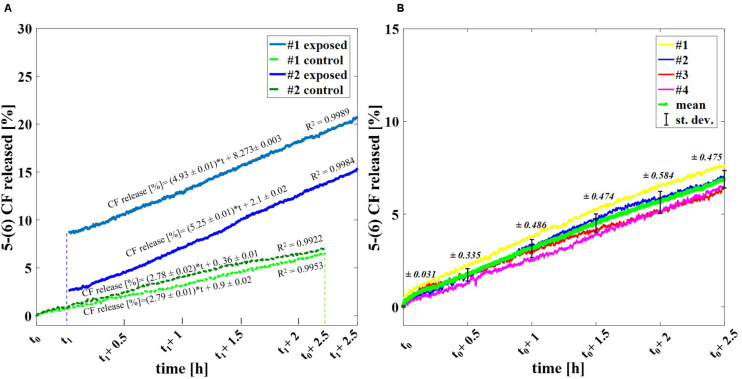
Profile in time of the experimental release of the fluorescent dye. **(A)** Percentage release of 5–(6) CF monitored in time up to 2.5 h for four different trials: two exposed samples using 2000 pulses and 2 Hz (dark and light blue) and two temperature control samples (dark and light green). **(B)** Spontaneous release of 5-(6) CF from liposomes due to the operative temperature of 37°C up to 2.5 h via spectrofluorimetry. A set of four different trails is reported (yellow, blue, red, magenta) with the calculated mean curve (green) and the standard deviation values as bars.

Figure 3A shows the percentage of the 5-(6) CF release at the starting time after exposure (t_1_ of Figure 1B, as described in section “Experimental protocol”) and at 2.5 h after t_1_ for exposed and control samples, comparing the experiments applying 2000 and 4000 ns pulses. The results are reported as the mean ± the standard deviation of the experiments and the statistical analysis shows that the difference between exposed and control samples is significant at all times with *p*-values < 0.001, thus confirming the effect of nsPEFs on the membrane of nanosized liposomal vesicles. The release of the fluorescent dye increases in time, as can be observed in Figure 3B reaching, after the 2.5 h of post-exposure, a value of 19.6 ± 3.3% confirming the role of electrical activation in the leakage of these vesicles. As it can be seen from Figure 3C, where the values of percentage of the 5-(6) CF release are reported for selected times in the case of 4000 pulses, the increase versus time of the control samples is almost constant between 0.8 and 1.1% every 0.5 h, while, after the first hour, the increase of the exposed sample rises significantly. For 4000 pulses applied, the difference between the exposed and the control starts slightly above 8% just at the end of the exposure and reaches around 13% at 2.5 h, while for the 2000 pulses the exposed versus control differences are slightly smaller but still statistically significant. Going into a deeper analysis in time, this effect is observed also in the case of 2000 pulses, looking at Figure 4, which reports the profile in time of the fluorescent dye release. Figure 4A reports the percentage release of 5–(6) CF monitored in time up to 2.5 h for four different trials: two exposed samples using 2000 pulses and 2 Hz (dark and light blue) and two temperature control samples (dark and light green). It is possible to observe the slopes of the curves of the two exposed samples 4.93 ± 0.01 and 5.25 ± 0.01 with respect to the slopes of the control ones, around 2.79 ± 0.01; this means that the electric pulse train is able to destabilize the membrane of the liposomes so that the dye was slowly released from the liposomes for hours after the end of the exposure. The spontaneous leakage from the vesicles due to the environmental temperature of 37°C causes an effect similar to the control (Figure 4B) determining a leakage of about 7% after 2.5 h, much smaller than the effect of the dye released from the exposed samples, ranging from 15% (2000 pulses) to 20% (4000 pulses). Finally, it is important to underline that the nsPEFs application did not lead to the liposomes rupture, as demonstrated by the hydrodynamic diameter data obtained before and after the pulse application, as reported in Supplementary Figure S1A. The data reported have been obtained using a pretreatment temperature of 37°C, considering the future application for the human body, but similar results have been obtained also using a pretreatment temperature of 25°C and then applying 2000 pulses in Supplementary Figure S1B.

### Characterization of the Experimental Bench

**FIGURE 5:**
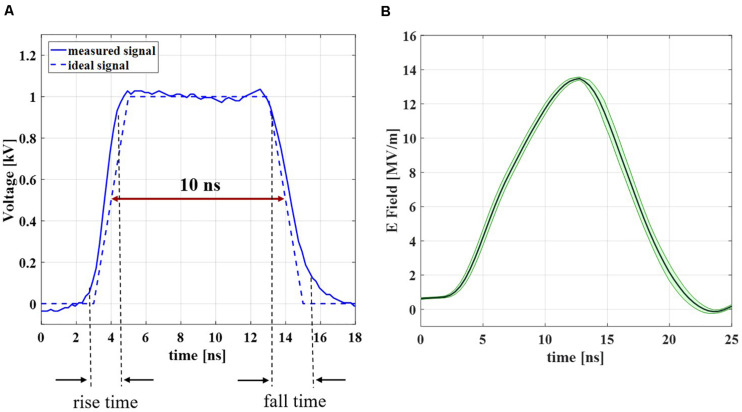
Experimental signal characterization results. **(A)** Measured signal at the generator (blue solid line) in comparison with the ideal signal (blue dashed line). **(B)** Measured electric field inside the 1 mm-gap cuvette during the exposure to a train of 4000 pulses with a PRF of 4 Hz. The results are reported as the mean curve (dark green solid line) ± standard deviation (light green shadow).

A full characterization of the experimental bench was performed in order to obtain complete reliability and control of the experimental data. As first step in the analysis of the experimental bench performance, the electrical signal delivered by the generator has been recorded. The result of a single recorded pulse is compared with the ideal trapezoidal pulse with a duration of 10 ns and rise and fall times of about 2 ns, as reported in Figure 5A. The two blue curves (solid and dashed, respectively for the experimental and ideal signal) are in good agreement with each other, with a cross-correlation coefficient = 0.9931. Figure 5B represents the mean electric field (dark green solid line) with the standard deviation (light green shadow) evaluated in the cuvette, measured from the voltage acquired with the two Testec HV probes (see inset Figure 1A) when the 4000 pulses train is applied on the liposomal suspension with a PRF of 4 Hz. The signal maintains a 10 ns duration and the very good repeatability of the pulses sequence is represented by the low standard deviation of the curve; the maximum intensity of the electric field in time inside the cuvette is about 14 MV/m, with 9 kV from the generator. More information about the parameter values in terms of mean and standard deviation of the acquired signals are reported in Supplementary Table S2.

**FIGURE 6:**
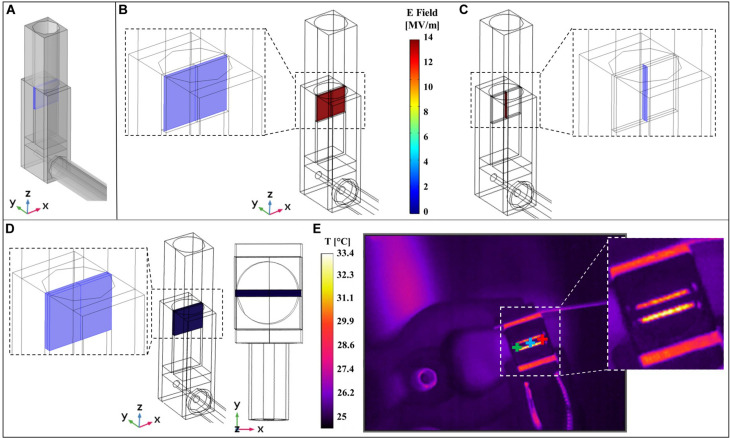
Comparison of simulated and experimental results. **(A)** 3D model of the experimental system used to perform multiphysics simulations. **(B)** Simulated electric field distribution inside the cuvette at *t* = 10 ns is homogeneous on the central slice parallel to the electrodes (the slice is represented in the left inset). **(C)** Simulated electric field distribution inside the cuvette at *t* = 10 ns is homogeneous on the central slice perpendicular to the electrodes (the slice is represented in the right inset). **(D)** Homogeneous simulated temperature distribution inside the volume of the solution sample placed in the cuvette, lateral and top view of the exposure system. **(E)** Image experimentally recorded with the thermal camera during the exposure to nsPEFs on the right, with three cursors point (green, blue, and red). A zoom of the image without cursors is reported in the right inset.

As reported in the inset of Figure 1A the nsPEFs signal is delivered to the sample using an *ad hoc* exposure system hosting the cuvette for electroporation. A numerical study has been carried out using COMSOL Multiphysics as explained in section “Materials and Methods” and considering a 3D model of the system, shown in Figure 6A where the cuvette, the transition from the coaxial cable to the brass parallel plates electrodes and the cable itself are modeled. Specifically, in Figures 6B,C are reported the electric field distributions inside the sample holder for two coordinate planes parallel and perpendicular to the electrodes, showing a homogeneous electric field of 14 MV/m at *t* = 10 ns, corresponding to the last time instant at the maximum intensity of the pulse.

Finally, the exposure system has been characterized in terms of temperature distribution comparing measurements and simulations. This is a crucial point since it is mandatory to monitor that the application of nsPEFs does not induce an increase of temperature in the sample thus becoming a confounding element in the interpretation of the final outcome. IR image of the system acquired with the thermal camera during the exposure is reported in Figure 6E, where three cursors point (green, blue, and red) are highlighted; in the inset is reported the temperature acquisition of the top of the cuvette hosting the solution. It is possible to observe that the temperature of the solution is around 25°C while electrodes reach a temperature of 33°C. The simulated 3D temperature distribution in the cuvette volume at 10 ns is reported in Figure 6D. As expected, the temperature distribution is homogeneous in the volume solution with a value comparable to the one acquired experimentally.

**FIGURE 7:**
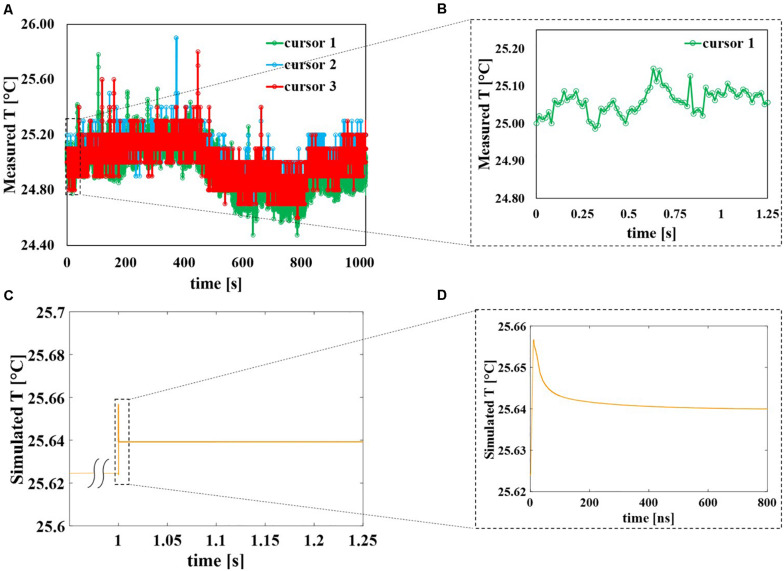
Evolution in time of the sample temperature, comparison between experimental and numerical data. **(A)** Temperature of the solution sample experimentally measured using the thermal camera with a frame frequency of 16.3 Hz during the entire exposure of about 17 min. The peaks represent the times during which the pulses are delivered to the solution and are reported as isolated events. **(B)** Zoom of temperature experimentally measured with cursor #1 from the beginning of the exposure up to 1.25 s corresponding to the initial 40 pulses. **(C)** Simulated temperature of the sample when the 40th pulse is applied to the sample, considering the worst-case scenario. **(D)** Zoom of the simulated temperature data from *t* = 0 ns of the 40th pulse application up to 800 ns.

The behavior of the three cursors in time is reported in Figure 7A for the whole duration of the exposure (around 17 min) with an acquisition frame frequency of 16.3 Hz. It is possible to observe that apart from higher peak values of almost 26°C the temperature inside the sample holder fluctuates between 24.6 and 25.2°C showing a negligible temperature increase induced by the application of the field. Even if during the 10 ns of the pulse application the electric field is extremely high (14 MV/m), during the remaining 250 ms the field is OFF, therefore eventual local peaks of the temperature in the solution drop rapidly and as a whole the temperature remains quite stable around 25°C. This can be seen in Figure 7B where a zoom of temperature data coming from cursor #1 up to 1.25 s is reported representing the first 40 pulses of the applied signal. Similarly, for the numerical study (see Figure 7C), the temperature of the external buffer is reported during the application of the 40th pulse, as the worst-case scenario. At 1 s the temperature profile (yellow curve) suddenly responds to the electric field stimulus; in the inset (Figure 7D) is reported a zoom of the temperature profile for the 40th pulse simulation: pulse ON at *t* = 0 ns, pulse OFF from *t* = 12 to 800 ns. The yellow curve increases of about 0.04°C and then it starts decreasing when the pulse is turned off, from *t* = 12 to 800 ns.

### Multiphysics Modeling of the Liposome Suspension

The main physical quantities involved in the interaction of electric field pulses and liposomes have been evaluated by means of a thorough numerical modeling. The simulations have been performed using up to 40 applied pulses with a PRF of 4 Hz and a maximum amplitude of about 14 MV/m, according to the experimental operating conditions and considering the computational costs. In fact already reproducing such conditions requires a high computational effort in terms of quasi-static solution of the electric currents module given that the total time duration of the simulation is almost 1 s with a variable time step, starting from 0.1 ns during pulse ON and gradually increasing to 0.2 ms during the pulse OFF time span.

**FIGURE 8:**
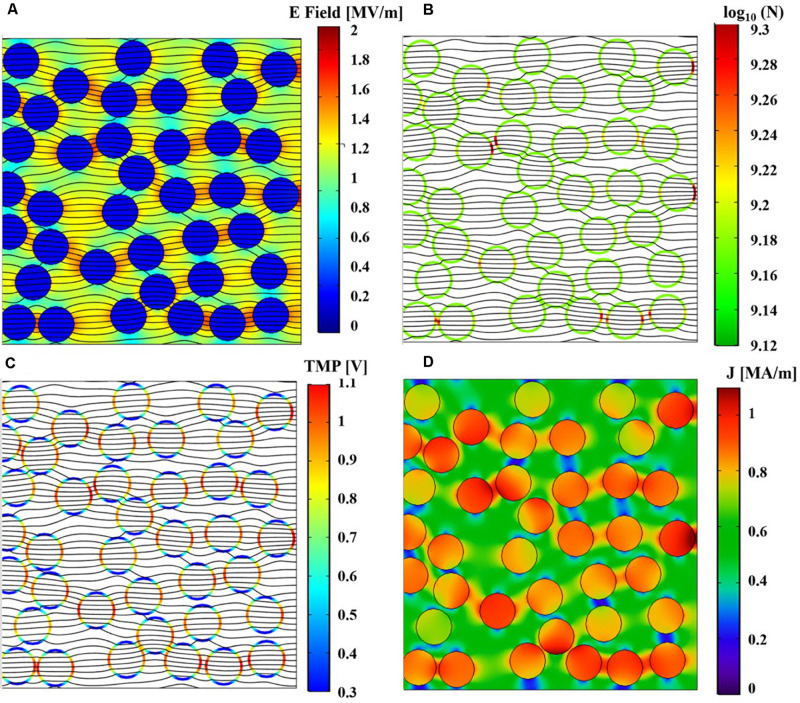
Spatial distributions of the simulated physical quantities on a section of the simulation box at *t* = 10 ns of the 40th pulse application. **(A)** Simulated electric field distribution together with the current density streamlines. **(B)** Simulated pore density distribution on liposome membranes together with the current density streamlines. **(C)** Simulated TMP distribution on liposome membranes together with the current density streamlines. **(D)** Spatial distribution of the simulated density current norm.

After the first 40 pulses, an indication of nsPEFs induced destabilization can be drawn by looking at the induced TMP, the pore density distribution on the liposome membranes, the temperature distribution and the changes of the electrical conductivity in time. Figure 8 shows a cut view from the whole initial model, reporting these quantities: E field (Figure 8A), pore density (Figure 8B), TMP (Figure 8C), and current density (Figure 8D) at the time *t* = 10 ns of the 40th applied pulse.

As expected, the electric field, normalized with respect to the maximum value applied, is higher outside the vesicles than inside, while the current density streamlines enter inside the vesicles due to the higher conductivity of the internal solution with respect to the external one, as also confirmed by the behavior of the current density norm in MA/m, of Figure 8D. The pore density spatial distribution on the liposome membranes shows how the effect of the electric field application is not uniform in the vesicle distribution; moreover, although somehow affected by the nsPEFs after the first 40 pulses, the poration density is still well below the threshold considered for an appreciable effect (see Figure 8B). Finally, the TMP distribution ranges from 0.3 to 1.1 V, showing higher values at the anode and cathode vesicles poles in line with the pore density distribution in terms of higher values (Figure 8C). As a whole it is possible to support the hypothesis that already after the first 40 pulses some slight effects in the coupling of the field are observable as the basis of the electroporation mechanism.

**FIGURE 9:**
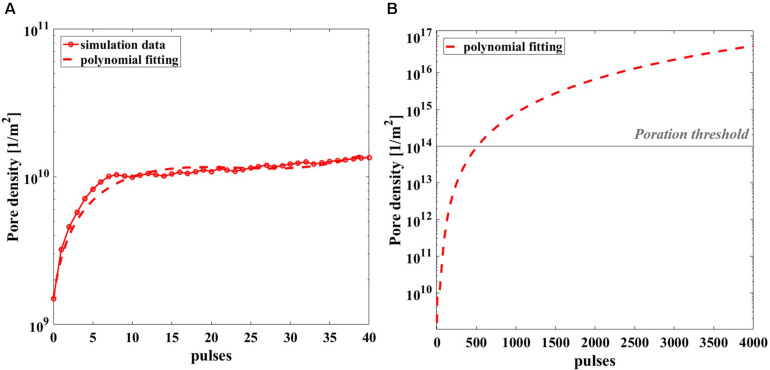
Simulated pore density profile in time of the most exposed liposome of the model during the nsPEFs exposure. **(A)** The pore density curve obtained from the simulation (solid line with empty red circle as marker) is reported with the third-grade polynomial fitting curve (dashed line) up to 40 pulses, *R*^2^ = 0.9207. **(B)** Polynomial fitting curve of the pore density curve extrapolated up to 4000 pulses.

Given the computational effort to reproduce the realistic exposure for 4 Hz repetition frequency, pore density in time has been calculated in the most exposed liposome of the model, for the 40 simulated pulses (red circles) and reported in Figure 9A, with a third-grade polynomial fitting curve (dashed line). The extrapolation of the polynomial curve up to 4000 pulses (Figure 9B) clearly reveals that starting from the 570th pulse the threshold for poration is overcome. In fact, once the well-known threshold value of 10^14^ m^–2^ for the poration of lipid membrane is reached (DeBruin and Krassowska, 1999; Retelj et al., 2013; Caramazza et al., 2019), the poration can start and give rise to a breakdown mechanism. It is assumed that for 2000 or 4000 pulses the threshold is highly exceeded hence, poration of the liposomes is definitively obtained, supporting the effect experimentally proven.

**FIGURE 10:**
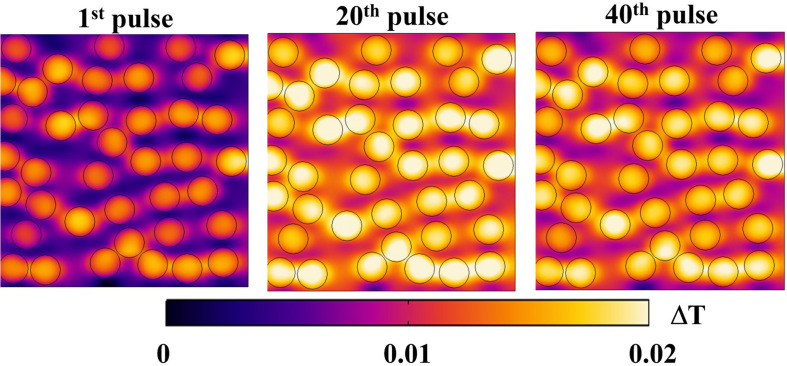
Temperature distribution on a section of the simulation box at 10 ns for three different pulses: 1st, 20th, and 40th, respectively, in the left, central and right panels. The distribution reported as ΔT = T–T_min_ for each pulse, changes during the exposure, maintaining the trend of higher values inside the vesicles and lower values outside.

Regarding a possible local thermal coupling, Figure 10 shows the microscopic temperature distribution on the same cut view of the previous electrical distributions, during the nsPEFs application. Higher values are localized inside the liposome vesicles and in the space between vesicles, showing both the vesicle-environment and the vesicle-vesicle interactions, but still in the order of a temperature difference of 0.02°C. It is argued that, despite the non-linear physics underlying the response of the system under investigation, the increasing number of pulses will not affect the thermal safety of electroporation process, as clearly indicated by the temperature measurements reported in Figure 7B.

## Discussion

In the past several decades, together with the improvement of nanotechnology and material science, various so-called stimuli responsive carriers have been successfully developed as promising vectors for drug delivery, i.e., so that the encapsulated therapeutics molecules could be released in a spatial or temporal (“*smart*”) delivery systems (Gu et al., 2018). Among the external stimuli, pulsed electric fields, as the ones used for electroporation of cells seem a promising technique to activate lipid vesicles carriers thus achieving a stimulus-sensitivity delivery of the payload encapsulated inside them. In particular the possibility to use nsPEFs, which have proven to electroporate intracellular organelles of cells, have suggested the idea to control the release from liposomes, at first electroporating liposomes once they are taken up by the cells (Perrier et al., 2017). A theoretical study already demonstrated the feasibility that internalized liposomes could be electroporated without affecting the cell viability (Retelj et al., 2013). Another approach could be to electroporate such lipid vesicles when they are in close proximity of the target cells, simultaneously electroporating both the cell and liposomes population, without compromising the cell viability. In fact, since nsPEFs are able to electroporate the cell membrane, the content released from the liposomes could be taken by the electroporated cells (Denzi et al., 2017b). However, the possibility of triggering the release of liposome content with nsPEFs remains up to now at the theoretical level, and the experimental feasibility of this approach has not been confirmed yet.

The experimental proof-of-concept presented in this paper supports the challenging issue that it is possible to electrically activate by means of nsPEFs (10 ns duration, 14 MV/m) the release of molecules entrapped in the interior of liposomes. Experimental data show that a train of pulses is required to obtain the effect; in particular 2000 pulses with a pulse repetition frequency of 2 Hz and 4000 pulses with a repetition frequency of 4 Hz. Moreover, the observed effect seems to be dose-dependent enhancing the release when increasing the number of pulses for the same duration of exposure. Temperature acquisitions during the exposure confirm that this kind of stimulus is non-thermal. The stability of the signal generated, in terms of repetition of electric pulses, guarantees that the exposure conditions are highly reproducible and well controlled.

An effort has been made to strengthen the experimental results, providing a theoretical investigation based on a multiphysics numerical simulation of the liposome solution exposed for the first time to a realistic train of electric pulses, characterized by the temporal multiscale of a very short pulse duration (10 ns) and a much longer time interval between pulses (250 ms). This allows a better comprehension of the field interaction with the target nanocarrier suspension. In particular from the numerical simulations related to the spatial distributions of the electrical quantities involved, we suggest that there is a consistent effect of focalization due to the close proximity of liposomes each other: the closer they are the higher such effect, including the local temperature around and inside the liposomes. In fact, even if, during the extremely short duration of the pulse, a local increase in temperature could happen due to the extremely high intensity of the electric field, this would rapidly diffuse during the longer period of quiescence of the signal (250 ms), so that the average temperature of the solution will remain at a constant temperature. Moreover having obtained a proper fitting of the liposome membrane pore density curve over the train of 40 simulated pulses, and extrapolating such curve in order to have a prediction of behavior up to the experimental condition of 2000 and 4000 pulses, one finds out that, in such conditions, the liposome membranes will be electroporated having overcome the threshold of 10^14^ m^–2^ for the electroporation of lipid membranes. This is in line with the experimental result which indicates that 2000 is the minimum number of pulses to obtain a significant release of the fluorescent dye from inside the nanosized liposomes exposed.

As a whole the significance of this work findings is related to the experimental validation of the possibility to on demand trigger the release from nanosized liposomes applying pulsed electric fields as such used for cells treatment. These outcomes open the way to the experimental application of liposomal drug delivery systems remotely activated by nsPEFs, previously studied only from a numerical point of view (Retelj et al., 2013; Denzi et al., 2017a, b). As a result of the lipid membrane destabilization, the fluorescent dye release of about 15–20% after a single treatment, is in line with literature on stimuli-sensitive drug delivery system activated by light or magnetic fields (Spera et al., 2015; Senapati et al., 2018; Liu and An, 2019). Thus, this relatively low percentage of release is useful thinking on the possibility to apply nsPEFs in a multi-dose manner. The numerical simulations confirm that liposome membrane electroporation could be achieved applying a sequence of nsPEFs with intensity in the order of MV/m and 10 ns duration. This numerical model could be a starting point to predict other exposure conditions.

## Conclusion

In this work, the experimental feasibility of electrical activation of liposome nanocarriers with nsPEFs has been proven by looking at the release of a fluorescent dye entrapped in the vesicles. Experiments were performed applying a train of nsPEFs (10 ns duration, 14 MV/m intensity and 2 and 4 Hz periodicity) on unilamellar liposome suspensions. Results show that liposome membranes are destabilized, allowing the release of transported compounds, in a statistically significant way up to almost 20%, and a dose-effect relationship is identified when a train of nsPEFs with higher PRF and number of pulses is applied. Multiphysics simulations have been performed in order to support the experimental data, studying the occurring mechanisms at a microscopic scale. A random distribution of liposomes was built, coupling three physical modules in order to consider the effect of nsPEFs interaction with liposome vesicles in terms of electromagnetic energy absorption, temperature distribution and pore density formation in time. The simulation results support the experiments giving an indication that not less than 500 pulses are needed to at least initiate the electroporation response. Therefore, here we prove the possibility to remotely activate nanosized liposomes with nsPEFs, with a comprehensive study in terms of both experiments and multiphysics simulations, giving a rational to design and perform an on-demand control of the release of transported compounds inside nanosized liposomes used as biocompatible reservoir, opening the way to future *in vitro* investigation.

## Data Availability Statement

All datasets presented in this study are included in the article/Supplementary Material.

## Author Contributions

FA, SP, and ML: conceptualization, funding acquisition, project administration, and supervision. LC and MN: data curation and validation. LC, FA, SP, and ML: formal analysis. LC, MN, AD, SP, and PP: investigation. FA, SP, ML, and AD: methodology. SP and PP: resources. LC, AD, FA, and ML: software. LC: visualization. All authors contributed writing, review and editing the article and approved the submitted version.

## Conflict of Interest

The authors declare that the research was conducted in the absence of any commercial or financial relationships that could be construed as a potential conflict of interest.
