# Supplementary material for: Proof-of-Concept of Electrical Activation of Liposome Nanocarriers: From Dry to Wet Experiments
Source: Front Bioeng Biotechnol. 2020 Jul 23;8:819. doi: 10.3389/fbioe.2020.00819 (PMC7390969; doi:10.3389/fbioe.2020.00819)
Supplement: Supplementary file 1 [file Data_Sheet_1.pdf]

Supplementary Material

Supplementary Table S1. Experimental data related to the characterization of the prepared liposome samples.

**Physico-chemical characterization of liposomes**	
**Sample**	Egg-PC
**Hydrodynamic diameter (nm)**	267.91 ± 1.71
**PdI**	0.214 ± 0.022
**ζ–potential (mV)**	(-) 24.12 ± 0.34
**Entrapment Efficiency (μL/mg)**	0.85 ± 0.04
**Structured phospholipid in vesicles (%)**	85.22 ± 1.21

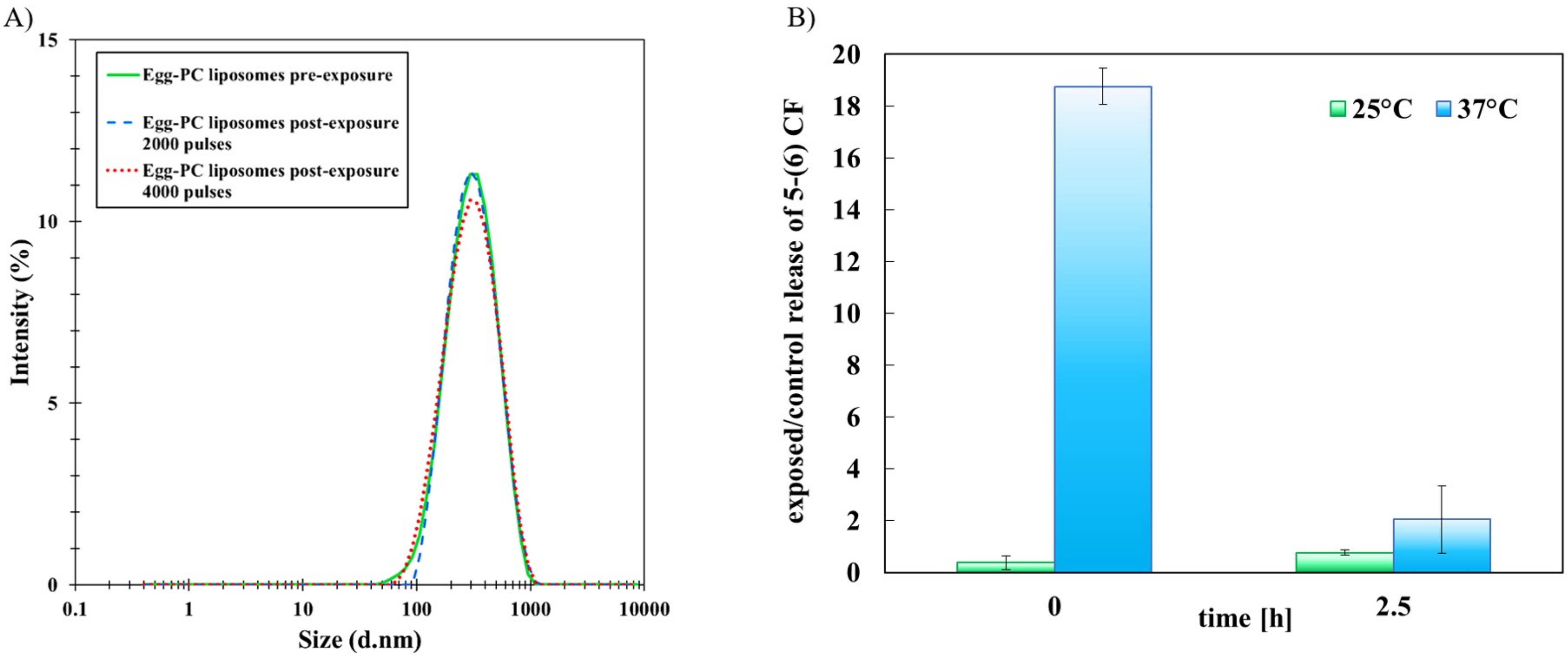

Supplementary Figure S1. (A) Experimental hydrodynamic diameter measurements carried out with Zetasizer Nano ZS90 for not-exposed liposomes (green curve), liposomes exposed to 2000 pulses (blue dotted curve), liposomes exposed to 4000 pulses (red dotted curve). **(B)** Experimental 5-(6) CF release in terms of ratio between exposed and control, comparing the results from two protocols: (i) 25 °C, 2000 pulses at 7 kV and 2 Hz (green); (ii) 37 °C, 2000 pulses at 9 kV and 2 Hz. The data are reported as the mean ± standard deviation. # 3 experiments performed for (i) and #8 experiments for (ii).

Supplementary Table S2. Experimental data acquired using a couple of HV-probes and the oscilloscope during the exposures. Inter-experiments data are related to the entire set of exposures, while intra-experiment data report the outcomes of the signal analysis performed starting from the train of pulses of one single exposure, as shown in Figure 5B in terms of electric field inside the cuvette.

**Inter-experiments signal acquisition**				
**# Pulses**	**Amplitude (kV)**	**Rise time (ns)**	**Fall time (ns)**	**FWHM (ns)**
2000	13.782 ± 0.018	6.551 ± 0.017	5.939 ± 0.147	10.991 ± 0.257
4000	13.427 ± 0.159	6.946 ± 0.048	6.032 ± 0.094	10.812 ± 0.076
**Intra-experiments signal acquisition**				
**# Pulses**	**Amplitude (kV)**	**Rise time (ns)**	**Fall time (ns)**	**FWHM (ns)**
4000	13.491 ± 0.083	7.209 ± 0.273	6.372 ± 0.224	10.461±0.355
